# Repellency of Eucalyptol and DEET Against *Triatoma infestans*, a Chagas Disease Vector: A Proof-of-Concept Study Under a Human Odor Background

**DOI:** 10.3390/insects17090881

**Published:** 2026-08-24

**Authors:** Leandro S. Wagner, Pablo G. Guerenstein

**Affiliations:** 1 Laboratory Control de Insectos Perjudiciales (Lab CONIPER), Centro de Investigación Científica y de Transferencia Tecnológica a la Producción (CICYTTP), Consejo Nacional de Investigaciones Científicas y Técnicas (CONICET), Gob. de la Prov. de Entre Ríos, Universidad Autónoma de Entre Ríos (UADER), Diamante CP 3105, Entre Ríos, Argentina; 2 Facultad de Ciencia y Tecnología, Universidad Autónoma de Entre Ríos (UADER), Diamante CP 3105, Entre Ríos, Argentina; 3 Facultad de Ingeniería, Universidad Nacional de Entre Ríos (UNER), Oro Verde CP 3100, Entre Ríos, Argentina

**Keywords:** repellent, kissing bug, biting inhibition, proboscis extension reflex, carvone, carvacrol, 1,8-cineole

## Abstract

Triatomines are vectors of Chagas disease. Control of *Triatoma infestans*, one of its main vectors, relies largely on the use of pyrethroid insecticides. However, due to resistance to them as well as environmental concerns, alternative control strategies are required. Here, we evaluated the volatile repellent activity of eucalyptol, carvacrol, carvone, and DEET (N, N-diethyl-3-methylbenzamide) under conditions that prevented the contact of the insects with the compounds, and under a human odor background, providing a realistic assessment of repellency for human protection. Importantly, eucalyptol and DEET were tested at low doses. Behavioral assays were conducted in a unidirectional olfactometer, mainly quantifying the following parameters: repellency coefficient (RC, 0 to 1 (1 = maximal repellency)) and biting inhibition. At 10 µL of pure compound, all chemicals induced strong and significant repellency (RC > 0.85). Eucalyptol and DEET remained highly effective at 1 µL (RC > 0.88) and continued to elicit significant repellency at 0.1 µL, whereas both compounds strongly inhibited biting behavior across the tested doses. Overall, our findings are a proof-of-concept suggesting that eucalyptol and DEET are highly effective volatile repellents and bite inhibitors against *T. infestans*, thus highlighting eucalyptol as a promising complementary alternative to DEET for human protection against Chagas disease.

## Simple Summary

Triatomines, also known as kissing bugs, are blood-feeding insects that transmit Chagas disease. Control of *Triatoma infestans*, one of the main vector species, relies largely on pyrethroid insecticides. However, insecticide resistance and environmental concerns highlight the need for additional strategies to protect people. Here, we evaluated the volatile repellent activity of eucalyptol, carvacrol, carvone, and DEET under conditions that prevented direct contact between the insects and the compounds, and in the presence of human odors, providing a realistic assessment of their potential for human protection. Moreover, eucalyptol and DEET were tested at very low doses. All four compounds strongly repelled the insects at the highest dose tested. Eucalyptol and DEET remained highly effective at lower doses and strongly reduced biting. Overall, our findings are a proof-of-concept suggesting that eucalyptol and DEET are highly effective repellents and bite inhibitors against *T. infestans*. These results highlight eucalyptol as a promising compound that could complement DEET in safer and more sustainable strategies for protecting people from Chagas disease.

## 1. Introduction

Triatomines (Hemiptera: Reduviidae) are hematophagous insects, commonly known as “kissing bugs”. These insects represent a major public health concern in the Americas due both to the allergic reactions caused by their bites and, primarily, to their role as vectors of the protozoan *Trypanosoma cruzi* (Chagas, 1909) (Kinetoplastida: Trypanosomatidae), the etiological agent of the American trypanosomiasis, also known as Chagas disease [1]. This disease, for which there is no vaccine available, is responsible for up to 50,000 deaths annually worldwide and, according to the World Health Organization (WHO), is a “neglected tropical disease” because it mainly affects low-income populations with limited access to healthcare services. Currently, an estimated 6 to 7 million people are infected, while approximately 75 million people are at risk of contracting the disease, mainly in Latin America [2,3].

In the southern cone of South America, *Triatoma infestans* (Klug, 1834) is the principal vector of Chagas disease. Its control relies largely on the application of pyrethroid insecticides, such as deltamethrin and cypermethrin, in domestic and peridomestic areas. Nevertheless, the continuous and widespread use of these compounds has led to the emergence of resistant populations in several regions [4,5,6,7]. In addition, intensive insecticide use can have detrimental effects on human health and the environment [8,9], emphasizing the need for alternative, safer, and more sustainable vector control strategies.

In this context, the use of spatial and topical repellents represents a promising strategy to reduce or prevent vector–host contacts, also mitigating the associated risks to human health and the environment [10]. DEET (N, N-diethyl-3-methylbenzamide) is widely regarded as the gold standard repellent against hematophagous arthropods [11]. Even when it has already been tested in *T. infestans*, its efficacy as a spatial repellent against this species warrants re-evaluation, as its repellent activity has been demonstrated primarily under conditions in which the insects can directly contact the compound and/or in the absence of host odor [12,13,14,15,16]. It should be mentioned that in those assays it was not possible to determine if the responses were due to the detection of DEET in the air or to irritant effects upon contact with it. This is particularly relevant because triatomines rely on volatile compounds for orientation and host location [17]. In contrast, when direct contact with the compound is prevented, and host odors are present, it has been suggested that repellency occurs only at very high, presumably toxic, doses [18,19]. A similar result has been reported for the kissing bug *Rhodnius prolixus*, in which only very high DEET doses produced significant repellency [19,20], whereas another study failed to detect repellency even at the highest dose tested [21].

Terpenes are secondary metabolites abundantly present in plant essential oils and are widely recognized for their repellent and insecticidal properties against insect pests, including species of both medical and veterinary importance [22,23]. Moreover, these compounds offer several advantages over conventional synthetic insecticides due to their favorable safety profile, allowing their use both on the skin and in the air [24,25]. To date, several terpenes have shown repellent activity against triatomines [26,27]. In *T. infestans*, carvacrol has exhibited strong repellent activity [27], whereas eucalyptol was effective only at high doses [13]. However, these studies have been carried out in the same experimental conditions as those with DEET (i.e., allowing direct contact with the test compound, and in the absence of human odor).

Therefore, we mainly asked if any of the tested compounds is capable of significantly reducing the approach of the insects towards the host. Thus, the main aim of this study was to evaluate the repellent activity of eucalyptol, carvacrol, carvone, and DEET against *T. infestans* under conditions that prevent direct contact with the test compound and with a human odor background. This should provide a proof-of-concept for their efficacy in preventing the approach of triatomines to humans.

## 2. Materials and Methods

### 2.1. Insects

Fourth-instar nymphs of *T. infestans* from laboratory colonies maintained at the Centro de Investigación Científica y de Transferencia Tecnológica a la Producción (CICYTTP, Diamante, Argentina) were used. The colony was originally established in 2011 from *T. cruzi*-free, insecticide-susceptible, wild populations collected in Chaco Province (northwestern Argentina), regularly provided by the Unidad Operativa de Vectores y Ambiente (UnOVE, CeNDIE-ANLIS Malbrán, Santa María de Punilla, Córdoba, Argentina). To maintain genetic diversity, the colony has been regularly supplemented with wild individuals from the same region approximately every two years. Insects were reared at 28 ± 2 °C and 55 ± 10% relative humidity under an artificial 12:12 h light:dark (L:D) photoperiod. Nymphs were fed biweekly on hens. For the experiments, individuals were separated from the colony immediately after molting and subjected to a starvation period of at least 30 days. Procedures were approved by the Committee of Ethics and Safety in Experimental Work of our institution (Expte. CEYSTE-CES-01339/2024, approved on 17 April 2024).

### 2.2. Behavioral Assays

All behavioral assays were conducted at the Laboratorio de Control de Insectos Perjudiciales (Lab CONIPER, CICYTTP, Diamante, Entre Ríos, Argentina) during the early scotophase at a temperature of 24.9 ± 0.7 °C and a relative humidity of 46 ± 9%, in a room lit with red light to minimize visual interference [28]. Experimental sessions were video-recorded using a Samsung Galaxy A12 smartphone (model SM-A125M; Samsung Electronics, Suwon, Gyeonggi Province, South Korea). Each insect was tested individually and used only once.

**Figure 1:**
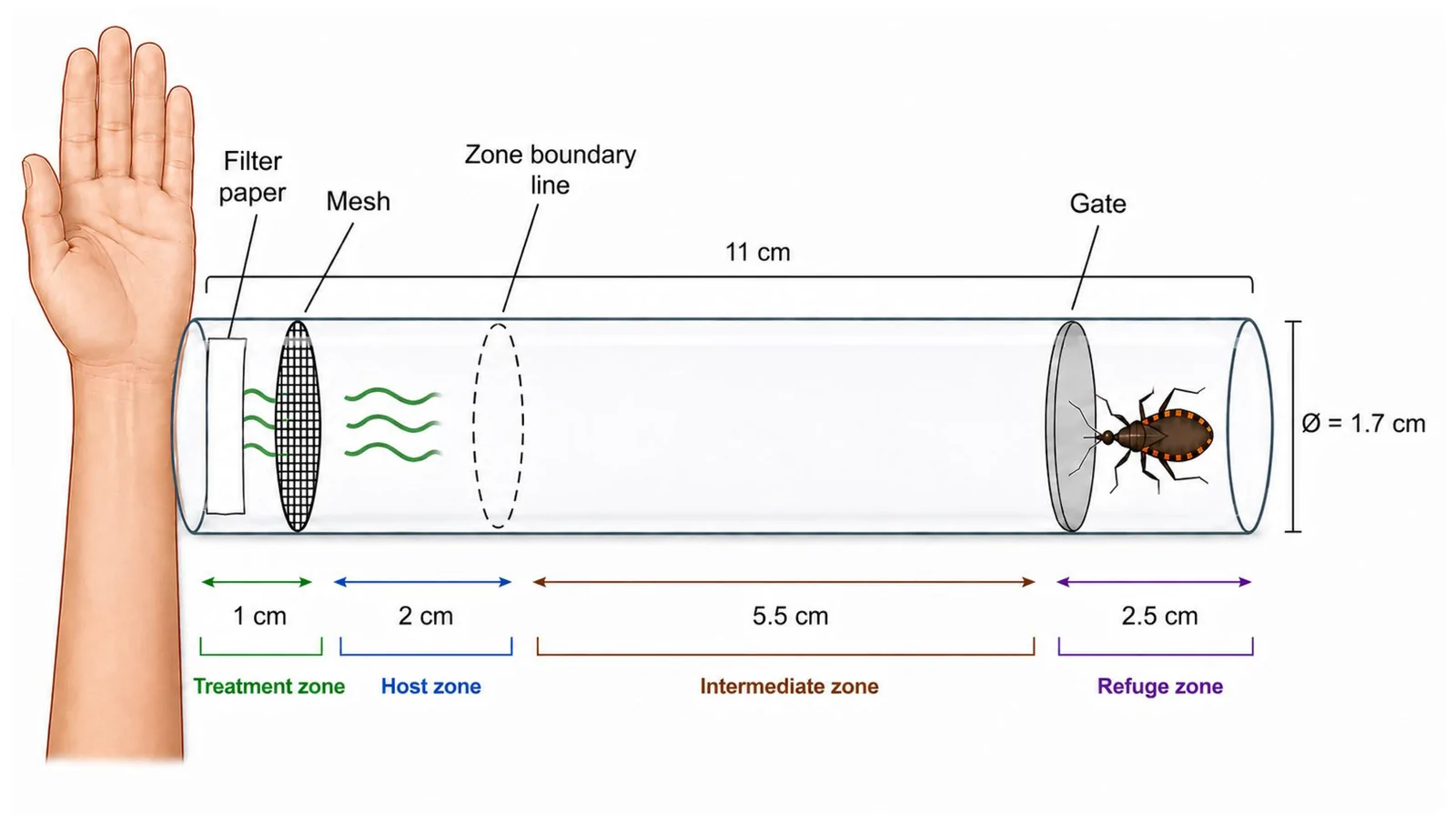
Unidirectional olfactometer used to evaluate the behavioral responses of *Triatoma infestans* to test compounds in the presence of host odors. The olfactometer consisted of a polystyrene tube divided into four zones: refuge, intermediate, host, and treatment. A sliding gate separated the refuge zone from the rest of the olfactometer. A mesh placed at the end of the host zone allowed the permeation of volatiles from the host and the test compounds while preventing the insects from biting the volunteer’s forearm.

Behavior was evaluated using a unidirectional olfactometer based on the design described by Ramirez et al. [19] (Figure 1). The olfactometer consisted of a polystyrene tube (11.0 cm × 1.7 cm; length × diameter) divided into four zones: refuge, intermediate, host and treatment (2.5, 5.5, 2 and 1 cm in length, respectively) and (1 cm). For each replicate, a single insect was placed in the refuge zone and allowed a 3 min acclimation period. The assay began with the opening of a sliding gate, allowing the insect to move freely between zones. Insects that did not move between zones during the 5 min assays were discarded and replaced (this represented < 2% of all insects tested). At the end of the host zone, a mesh was placed to prevent the insect from biting the volunteer’s forearm. To standardize the volatile organic compound (VOC) profile, the same volunteer was used throughout the study. The volunteer was a 40-year-old male who, for the period of assays, followed a strict lifestyle protocol, avoiding alcohol consumption, spicy foods, and the use of perfumed soaps and cosmetics. Also, the volunteer was a non-smoker, had no chronic illnesses, and did not take any medication.

For treatments with chemical compounds, a 1.0 cm × 3.0 cm filter paper strip was impregnated with a certain volume of a pure terpene ((R)-(−)-carvone, eucalyptol (1,8-cineole), or carvacrol; purity ≥ 98%, Merck-Sigma-Aldrich, Ciudad Autónoma de Buenos Aires, Argentina) or pure DEET (purity ≥ 97%, Sigma-Aldrich, Ciudad Autónoma de Buenos Aires, Argentina). A 10 µL dose was initially chosen based on the literature [19,29]. For (negative) control treatments, a filter paper strip without any compound was used. Filter paper strips were placed in the olfactometer between the volunteer’s forearm and the mesh screen, ensuring that no direct contact occurred between the chemical compounds and either the host’s skin or the insects.

Two experimental series were conducted. The first series compared: (i) 10 µL terpene versus control, (ii) 10 µL DEET versus control, and (iii) control versus control. The second series evaluated eucalyptol and DEET at lower volumes (0.1 µL and 1 µL). On each experimental day, three control and three treatment replicates were conducted. The order of stimulus presentation (control or treatment) and the selection of the host’s forearm were randomly assigned each day.

### 2.3. Data Analysis

Sample size (15 replicates per treatment) was determined based on an a priori Monte Carlo power analysis [30,31] (Supplementary Material, Figure S1). For each replicate, the following response variables were recorded: (i) time spent in the host zone, to assess repellency; (ii) number of biting attempts (as, occurrences of the proboscis extension reflex (PER) in the host zone), to assess biting inhibition; and (iii) total number of zone crossings by each insect, to assess locomotor activity.

The repellency coefficient (RC) was calculated as RC = (Tt − TA)/Tt, where Tt represents the total experimental time (5 min) and TA is the time the nymph spent in the host zone [32]. RC ranges from 0 to 1, with 1 indicating maximal repellency and 0 indicating maximal attraction. RC was analyzed using generalized linear mixed models (GLMMs) with a beta distribution and logit link function. Because the beta distribution only accommodates values greater than 0 and less than 1, RC values were transformed following the Smithson and Verkuilen [33] correction prior to analysis. This transformation was necessary because some observations reached the upper boundary value (RC = 1). RC estimates were expressed as estimated marginal means (EMMs) and their corresponding 95% confidence intervals. Biting inhibition was assessed using PER as a binary response (presence/absence of proboscis extension) and analyzed using GLMMs with a binomial distribution and logit link function. PER responses were expressed as estimated marginal probabilities of PER occurrence and their corresponding 95% confidence intervals. Locomotor activity was analyzed using GLMMs with a negative binomial distribution (nbinom2) and log link function. Responses were expressed as EMMs and their corresponding 95% confidence intervals on the response scale (number of zone crossings).

In all models, treatment was considered as a fixed effect and experimental day as a random effect to account for temporal variability. Each model was fitted using the glmmTMB package (v1.1.14), and comparisons between each compound and its corresponding control were evaluated within the model. EMMs and their 95% confidence intervals were obtained from the fitted models using the emmeans package (v2.0.2). Model assumptions were assessed through simulated residual diagnostics implemented in the DHARMa package (v0.5.0) [34]. Figures were generated using the ggplot2 package (v4.0.2). All analyses were conducted in R (RStudio v.2026.1.2.418; Posit Software, PBC, Boston, MA, USA) with a significance level of α = 0.05.

## 3. Results

### 3.1. Repellent Activity

**Figure 2:**
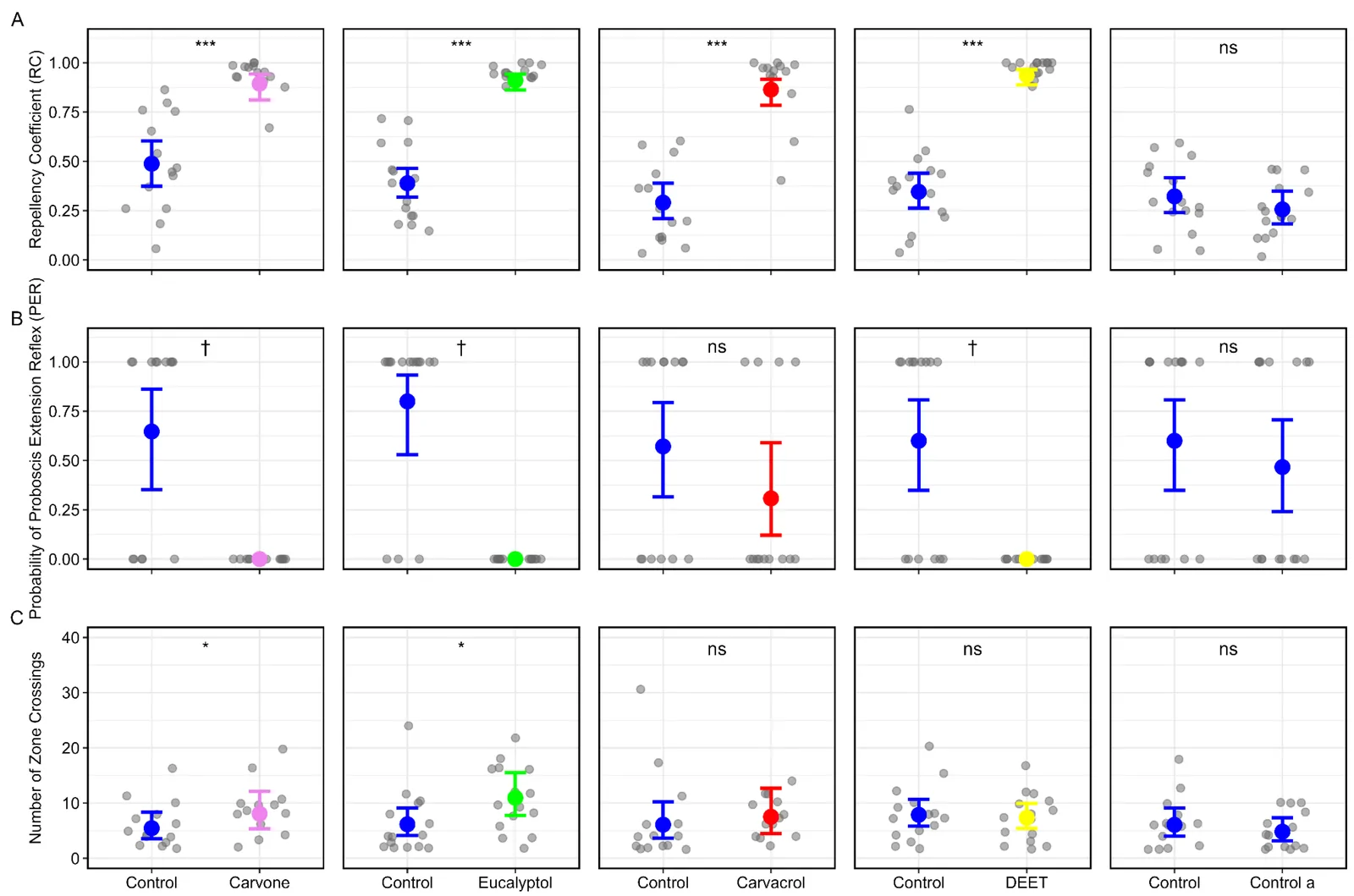
Behavioral responses of fourth-instar nymphs of *Triatoma infestans* exposed to carvone, eucalyptol, carvacrol, and DEET at a 10 µL dose. (**A**) Repellency coefficient (RC). (**B**) Biting inhibition, as the occurrence of the proboscis extension reflex (PER). (**C**) Locomotor activity, as the number of zone crossings. RC and locomotor activity are presented as estimated marginal means (EMMs), whereas PER is presented as estimated marginal probabilities. Error bars indicate 95% confidence intervals. Points represent individual replicates (N = 15 insects per treatment). Statistical differences between control and treatments are indicated as: ns, not significant; * *p* < 0.05; *** *p* < 0.001; † complete separation of the data, as all insects exposed to carvone, eucalyptol, and DEET showed 0% PER response.

Carvone, eucalyptol, carvacrol, and DEET, at 10 µL, exhibited strong repellency against *T. infestans*, significantly greater than that observed in their respective controls (GLMM beta: carvone: z = 6.16; eucalyptol: z = 9.78; carvacrol: z = 7.29; DEET: z = 9.81; all *p* < 0.001; N = 15). High repellency coefficients (RC > 0.85) were observed for all treatments. Thus, mean RC values (95% CI) were 0.894 (0.812–0.943) for carvone, 0.910 (0.862–0.942) for eucalyptol, 0.864 (0.785–0.917) for carvacrol, and 0.937 (0.887–0.965) for DEET (Figure 2A). No significant differences were detected between the negative control treatments without compounds (z = −1.28, *p* = 0.199; N = 15), and their RC values did not exceed 0.32 (Figure 2A).

**Figure 3:**
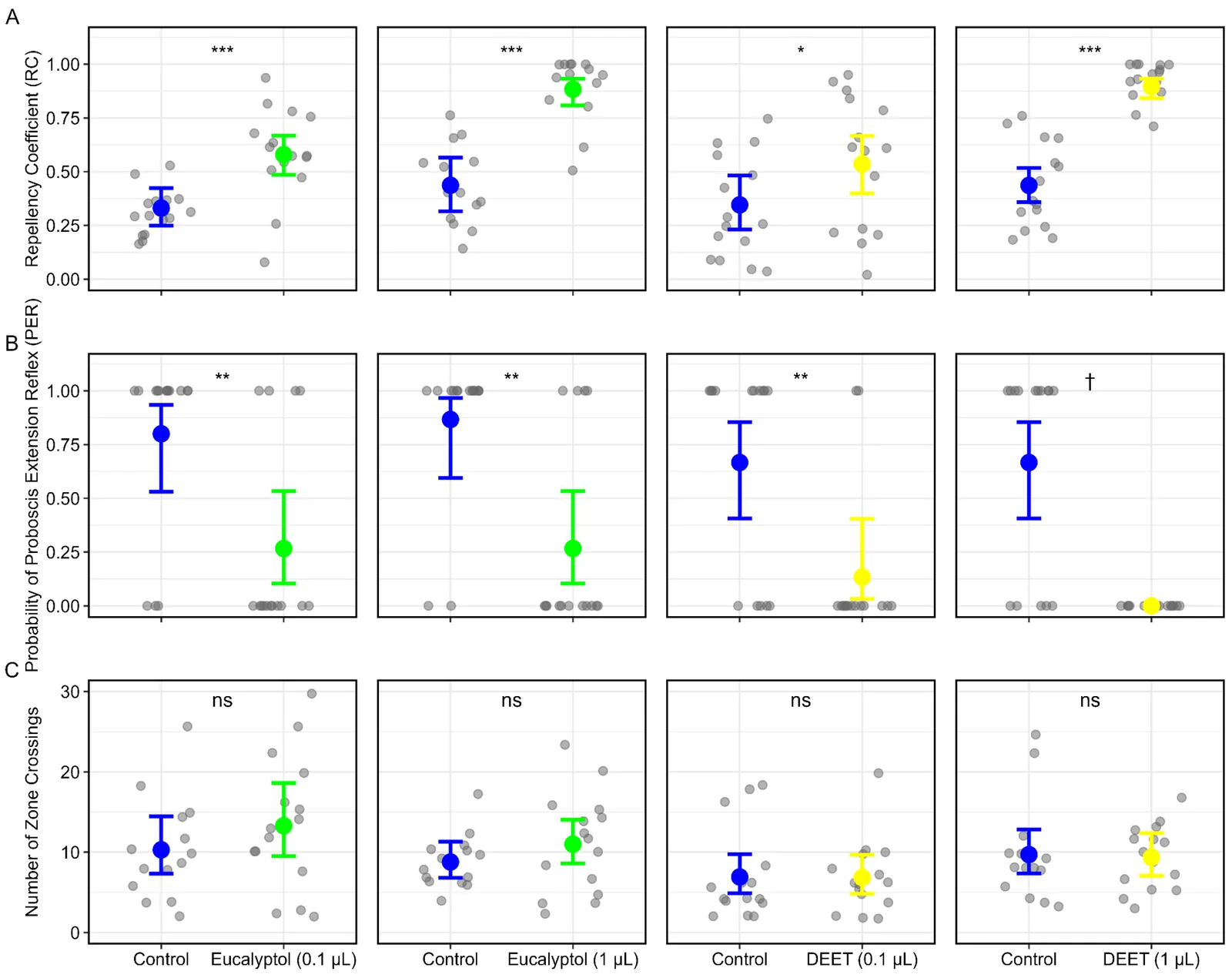
Behavioral responses of fourth-instar nymphs of *Triatoma infestans* exposed to eucalyptol and DEET at 0.1 and 1 µL doses. (**A**) Repellency coefficient (RC). (**B**) Proboscis extension reflex (PER). (**C**) Locomotor activity, as the number of zone crossings. RC and locomotor activity are presented as estimated marginal means (EMMs), whereas PER is presented as estimated marginal probabilities. Error bars indicate 95% confidence intervals. Points represent individual replicates (N = 15 insects per treatment). Statistical differences between control and treatments are indicated as: ns, not significant; * *p* < 0.05; ** *p* < 0.01; *** *p* < 0.001; † complete separation of the data because all insects exposed to DEET at 1 µL showed 0% PER response.

Eucalyptol and DEET, at 1 µL, exhibited strong repellent activity significantly different from their respective controls (GLMM beta: eucalyptol: z = 8.35; DEET: z = 8.02; both *p* < 0.001; N = 15). Thus, eucalyptol reached an RC value of 0.88 (95% CI: 0.81–0.93), whereas DEET showed an RC of 0.90 (95% CI: 0.84–0.93) (Figure 3A; Supplementary Material, Video S1). At the lowest dose tested (0.1 µL), eucalyptol and DEET also produced significant repellent responses relative to their controls (GLMM beta: eucalyptol, z = 4.14; *p* < 0.001; DEET, z = 2.17; *p* = 0.030; N = 15), although the magnitude of the repellency was substantially lower, with RC values of 0.58 (95% CI: 0.49–0.67) and 0.54 (95% CI: 0.40–0.67), respectively (Figure 3A).

### 3.2. Biting Inhibition

Carvone, eucalyptol, and DEET, at 10 µL, completely suppressed the proboscis extension reflex (PER) in all tested insects, whereas estimated PER probabilities in the corresponding control groups exceeded 0.60 in all cases (N = 15). This complete data separation precluded formal estimation of the logistic model parameters. In contrast, carvacrol did not significantly affect PER (GLMM binomial: z = −1.36; *p* = 0.174; N = 15), although the estimated PER probability was lower in exposed insects (0.31; 95% CI: 0.12–0.59) than in the corresponding control group (0.57; 95% CI: 0.32–0.79) (Figure 2B). No significant differences were detected between control groups (GLMM binomial: z = −0.73; *p* = 0.466; N = 15) (Figure 2B).

Eucalyptol significantly reduced PER at both 1 and 0.1 µL compared with the corresponding controls (GLMM binomial: z = −3.01; *p* = 0.003; and z = −2.75; *p* = 0.006, respectively; N = 15). The estimated PER probability was 0.27 (95% CI: 0.10–0.53) at both doses (Figure 3B). DEET at 1 µL completely suppressed PER, precluding formal estimation of the logistic model parameters (N = 15). At 0.1 µL, DEET significantly reduced PER relative to the corresponding control (GLMM binomial: z = −2.74; *p* = 0.006; N = 15), with an estimated PER probability of 0.13 (95% CI: 0.03–0.41) (Figure 3B).

### 3.3. Locomotor Activity

Carvone and eucalyptol at 10 µL significantly increased the locomotor activity of the insects (GLMM negative binomial: z = −2.33; *p* = 0.020; and z = −2.25; *p* = 0.025; N = 15, respectively), with estimated mean numbers of zone crossings of 8.1 (95% CI: 5.4–12.1) for carvone and 11.0 (95% CI: 7.7–15.5) for eucalyptol. In contrast, no significant effects on locomotor activity were detected for carvacrol (GLMM negative binomial: z = −0.84; *p* = 0.403; N = 15) or DEET (GLMM negative binomial: z = 0.32; *p* = 0.749; N = 15) (Figure 2C). Also, no significant differences were detected between control groups (GLMM negative binomial: z = 1.12; *p* = 0.263; N = 15) (Figure 2C).

At 1 and 0.1 µL, neither eucalyptol (GLMM negative binomial: z = 1.33; *p* = 0.184; and z = 1.17; *p* = 0.242; N = 15, respectively) nor DEET (GLMM negative binomial: z = −0.20; *p* = 0.838; and z = −0.04; *p* = 0.967; N = 15, respectively) significantly affected the locomotor activity (Figure 3C).

## 4. Discussion

In its broadest definition, a repellent is any chemical compound, mixture of compounds, or stimulus that reduces or prevents an insect’s ability to search for, locate, recognize, approach, and/or successfully feed on its host [35,36,37,38,39]. Under this definition, and based on observed insect behavior, repellency can be categorized into five types, including true repellency, odor masking, contact irritancy, visual masking, and antifeedant [36]. In our experimental setup, the insects could not contact the tested compounds, and we suggest that DEET, eucalyptol, carvone, and carvacrol acted as volatile repellents. Moreover, once the insects started to approach the stimulus source, they usually moved away from it; we suggest that those compounds acted as true repellents, at least at short range. Apart from evaluating the repellent activity of the test compounds, we also studied additional behavioral parameters, including biting attempts and locomotor activity.

At the highest dose tested (10 µL of pure compound), DEET, eucalyptol, and carvone, in their vapor phase, exhibited similarly high levels of repellency and biting inhibition, whereas only carvone and eucalyptol increased the locomotor activity of the insects. We also tested DEET and eucalyptol at lower doses (0,1 and 1 µL of pure compound). Eucalyptol was selected over carvone because of its wide availability and low cost, with prices ranging from 15 to 30 USD/kg [40]. Moreover, costs could be further reduced by using eucalyptus essential oil, which contains up to 85% eucalyptol and costs less than 15 USD/kg [41,42], although its repellent activity should be experimentally confirmed. DEET, in turn, was selected because evidence of its repellent efficacy against *T. infestans* in the presence of human odor is largely based on a single study conducted at just a very high dose [19].

### 4.1. Repellent Effect of Eucalyptol and DEET at Low Doses

Eucalyptol and DEET, in their vapor phase, not only acted as effective repellents at a 10 µL dose, but also maintained a virtually unchanged repellency at a tenfold lower dose (1 µL). Furthermore, although the repellent effect of both compounds was markedly reduced at 0.1 µL, this dose was still sufficient to significantly decrease the tendency of the insects to stay in the host zone, especially in the case of eucalyptol.

Contrary to our results, and under experimental conditions characterized by direct contact of the insects with the test compound and absence of host odors, previous studies have found that eucalyptol exhibits very weak repellency, with significant effects only at high doses, reaching RC values close to 0.80 just at a dose of 3900 µg/cm^2^ in first-instar nymphs of *T. infestans* [13]. In that study, DEET achieved comparable repellency at approximately a 10-fold lower dose. In contrast, carvacrol, under the same experimental conditions, has been reported to induce strong repellency (RC > 0.80) against *T. infestans* even at low doses (39 µg/cm^2^) [43], whereas DEET required approximately a 10-fold higher dose to reach a similar level of repellency [13,43].

To date, only one study has evaluated the repellent activity of terpene-rich essential oils against triatomines in the presence of a background of human odor and excluding direct contact with the treatments. That study suggested that a 1:1:1 mixture of peppermint, lavender, and citronella essential oils at a dose of 10 µL is an effective repellent against fifth-instar nymphs of *Triatoma pallidipennis*, *T. infestans*, and *R. prolixus*, reporting estimated repellency coefficients (RC) greater than 0.80 [29]. However, no study has tested the repellent effect of single terpenes against triatomines in such experimental conditions.

Our findings suggest that DEET acts as a potent human volatile repellent against *T. infestans* through olfactory-mediated mechanisms, exhibiting substantially higher repellent activity than previously reported, especially at relatively low doses. Thus, to our knowledge, DEET repellency against *T. infestans* under our experimental conditions (i.e., with human odor background and detection of tested compounds just in the air) has been documented in only one study, in which a single high dose of 45 µL (approximately 450-fold higher than the lowest effective dose in our study) elicited significant repellency [19]. It should be mentioned that in that work, low doses of DEET were not tested in *T. infestans* because, under similar experimental conditions, the kissing bug *R. prolixus* showed significant repellency only at high doses [19,20], while no repellency was detected at doses close to 0.5 µL [21]. It should be mentioned that in *T. pallidipennis*, 10 µL of DEET induced an estimated repellency coefficient greater than 0.7 [29].

### 4.2. Biting Inhibition Induced by Terpenes and DEET

As mentioned above, carvone, eucalyptol, and DEET, in their vapor phase, completely inhibited biting attempts in *T. infestans* at the highest dose tested (10 µL), including in insects that briefly explored the host zone, whereas carvacrol did not inhibit biting attempts, despite evoking strong repellency at this dose and having been previously reported as an effective repellent against *T. infestans* [13]. Moreover, at lower doses (0.1 and 1 µL), eucalyptol markedly reduced the PER, although it did not completely abolish it, whereas DEET still produced complete inhibition at 1 µL. To date, no bite-inhibitory activity induced by terpenes or essential oils has been reported in triatomines. In contrast, previous studies suggested that DEET, in its vapor phase, has some, albeit very limited, efficacy as a bite inhibitor. Thus, in nymphs of *R. prolixus*, even very high doses (estimated ≈ 45 µL under comparable conditions) failed to completely inhibit feeding attempts [20]. In assays in which the DEET was applied to a mesh on pigeons, high doses of DEET (estimated ≈ 5 and 50 µL) failed to completely suppress feeding in nymphs of *T. infestans* [12]. In summary, our results indicate that carvone, eucalyptol, and particularly DEET, in their vapor phase, exhibit high bite-inhibitory efficacy in *T. infestans*.

### 4.3. Effects of Terpenes and DEET on Locomotor Activity

Increased locomotor activity (hyperactivity), without attraction or repellency responses, lacks directionality [12,13,27]. In triatomines, this phenomenon has practical relevance, because applying a chemical in a dwelling that evokes this response may induce insects to leave their shelters, thereby facilitating their detection [44]. Nevertheless, in our experimental setup we considered the possibility that locomotor hyperactivation could imply a reduction in repellency coefficient (RC) values, since a greater number of crossings between the olfactometer zones could increase the time spent in the stimulus zone. Therefore, evaluating this parameter was necessary to properly interpret the repellency response.

Only carvone and eucalyptol, and only at the highest dose tested, induced a slight increase in locomotor activity. At lower doses, eucalyptol did not produce significant increases in locomotion. The low or absent hyperactivity observed in most assays supports the interpretation of the repellency results, suggesting that the responses obtained were not affected by high locomotor activity. According to the literature, a high dose of eucalyptol did not alter locomotor activity in fifth-instar nymphs of *T. infestans* [43], whereas the same dose did induce hyperactivity in first-instar nymphs [13]. These discrepancies may reflect a greater susceptibility of juvenile stages to chemical compounds such as terpenes.

## 5. Conclusions

Our results mainly suggest that eucalyptol and DEET are highly effective human volatile repellents and bite inhibitors against *T. infestans*, even at doses much lower than those used in the literature. This result was unexpected considering the well-established low sensitivity of *R. prolixus* to DEET in its vapor phase [19,20,21], whereas it was also believed that *T. infestans* exhibits low sensitivity to that compound [16,20], especially in its vapor phase [19]. In addition, in a previous study, *T. infestans* showed low sensitivity to eucalyptol [13]. Therefore, the results obtained in this study show that *T. infestans* can be repelled effectively with compounds that are readily available and, in the case of compounds like eucalyptol, are also cheap.

It is well established that the odor background influences insect responses to odor stimuli [39]. In this study, the odor background consisted of human odor. However, only one human subject was used. Therefore, it would be important to extend the study using human subjects of different genetic backgrounds, ages, sexes, and diets. This would increase the generalizability of the results, although the high variability of individual human odor profiles [45,46] would make a comprehensive generalization difficult. In any case, the results obtained in this work represent a proof-of-concept for the importance of testing repellents under a real-life odor context, and also suggest the potential efficacy of eucalyptol and DEET as volatile repellents for human use against *T. infestans*.

Our findings highlight the potential of eucalyptol as a complementary compound to DEET for the development of improved repellent strategies against this Chagas disease vector. A limitation of this study relates to the use of pure compounds for tests. However, neither eucalyptol nor DEET are intended for direct application to the skin in their pure form. Future studies should focus on developing stable, controlled-release formulations of diluted eucalyptol and/or DEET, such as micro- or nanoencapsulation systems, to prolong repellent activity and reduce skin permeation. In addition, human toxicity and field-efficacy evaluations should be performed [47].

## Data Availability

The original contributions presented in this study are included in the article. Further inquiries can be directed to the corresponding author.

## Supplementary Materials

The following supporting information can be downloaded at: https://www.mdpi.com/article/10.3390/insects17090881/s1. Figure S1: Sample size and power analysis. Video S1: Repellency of eucalyptol and DEET to *Triatoma infestans* under a background of human odors. This is available online at https://zenodo.org/records/21726333 (accessed on 31 July 2026).
