# Supplementary material for: Repellency of Eucalyptol and DEET Against Triatoma infestans, a Chagas Disease Vector: A Proof-of-Concept Study Under a Human Odor Background
Source: Insects. 2026 Aug 24;17(9):881. doi: 10.3390/insects17090881 (PMC13607502; doi:10.3390/insects17090881)
Supplement: Supplementary file 1 [file insects-17-00881-s001.zip › insects-4437884-supplementary.pdf]

Figure S1: Sample size (15 replicates per treatment) was determined a priori using the simulation-based power analysis for GLMM (Monte Carlo). Simulations were performed under predefined model parameters assuming a beta-distributed response (φ = 10) while other parameters consisted of RC mean responses of 0.40 and 0.70 for the control and treatment groups, respectively, moderate between-day variability (SD = 0.30), a significance level of α = 0.05, and 1,000 Monte Carlo simulations. Fifteen replicates per treatment achieved a statistical power of 0.97 and were therefore considered sufficient for the study.

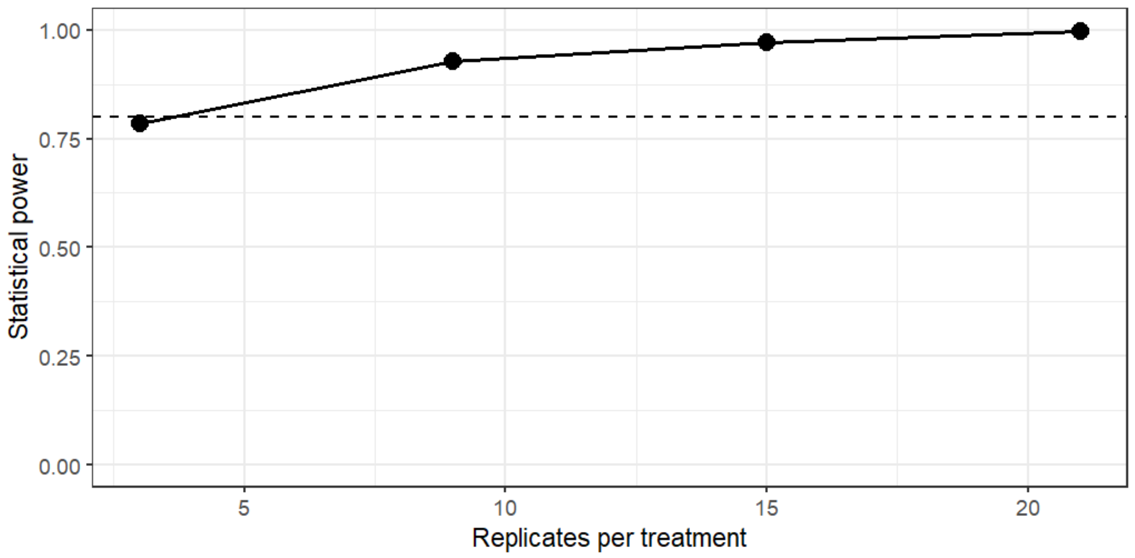


```
> ##############################################################
> # RESULTS
> ##############################################################
>
> print(results)
  replicate DAY power
1                                3     1 0.784
2                                9     3 0.927
3                               15     5 0.972
4                               21     7 0.997
>
```
